# Contraceptive needs and fertility intentions of women with breast cancer in Cape Town, South Africa: a qualitative study

**DOI:** 10.1186/s12905-020-01094-3

**Published:** 2020-10-06

**Authors:** Jane Harries, Deborah Constant, Lydia Cairncross, Jennifer Moodley

**Affiliations:** 1 Women’s Health Research Unit, School of Public Health and Family Medicine, University of Cape Town, Cape Town, South Africa; 2 Department of Surgery, University of Cape Town, Cape Town, South Africa; 3 Cancer Research Initiative, Faculty of Health Sciences, University of Cape Town, Anzio Road, Observatory, Cape Town, 7925 South Africa; 4 SAMRC Gynaecology Cancer Research Centre, Faculty of Health Sciences, University of Cape Town, Anzio Road. Observatory, Cape Town, 7925 South Africa

**Keywords:** Breast cancer, Contraception, Fertility intentions, Fertility preservation, South Africa, Qualitative methods

## Abstract

**Background:**

No known studies have been undertaken in South Africa exploring the contraceptive and fertility needs and preferences of women of reproductive age (18–49) diagnosed with breast cancer. This study set out to understand the contraceptive needs and fertility intentions of women with breast cancer in Cape Town, South Africa.

**Methods:**

Qualitative in-depth interviews were conducted with 24 women diagnosed with breast cancer and 4 health care providers at a tertiary hospital in Cape Town, South Africa. We explored contraceptive use prior to diagnosis; the impact of breast cancer on future fertility intentions and contraceptive use; understanding of suitable contraceptive methods during and after treatment and women’s fertility related counseling needs during their continuum of care. Data were analysed using a thematic analysis approach.

**Results:**

Since being diagnosed with breast cancer, of those women using a contraceptive method, the non-hormonal intrauterine device (IUD) was the most commonly used method. However, women reported receiving limited information from health care providers about contraceptive use and future fertility planning post treatment when fertility desires might change. Many women reported limited information received from healthcare providers about the impact of cancer treatment on their future fertility. Most women did not receive information around fertility preservation options, and few were familiar with the concept. Providers focus was more on preventing pregnancy during treatment and ensuring a patient was on a non-hormonal contraceptive method. Providers supported a more holistic, multidisciplinary approach to breast cancer patient’s contraceptive and future fertility needs.

**Conclusions:**

Limited contraceptive and future fertility counseling were reported by women despite many women being provided with the IUD. There is a need for improved information and counseling regarding the impact of treatment on contraceptive and fertility options. It is important that cancer care providers provide timely information regarding fertility options and communicate with patients about their fertility concerns prior to treatment and throughout the course of survivorship. The development of evidence-based information tools to enhance patient-provider communication and counseling could address knowledge gaps.

## Background

Breast cancer remains the commonest cancer among women globally with about 2.1 million newly diagnosed female breast cancer cases in 2018 [1]. In South Africa, breast cancer is the commonest form of cancer among women, with an age-standardised incidence rate of 35.95 per 100,000 women and a major cause of cancer mortality [2]. South Africa does not have a national mammography screening programme. Typically, women with breast symptoms self-present to primary healthcare facilities and are referred to secondary or tertiary level health facilities for further investigation, treatment and care [3].

Breast cancer is an important women’s health issue, and premenopausal women with breast cancer comprise a special subgroup with unique concerns regarding contraception, early menopause, infertility, and future fertility options [4–7]. Despite women’s need for information on these issues their levels of knowledge are considered lacking compounded by limited fertility and contraceptive counseling available to women, especially younger women [4, 7–10].

Contraception is challenging for women with cancer, particularly those with breast cancer, who are limited to long-acting non-hormonal methods, the copper T380A intrauterine device (IUD), as hormonal contraception is contraindicated and discontinued in women with breast cancer [10–13]. Contraceptive counseling in breast cancer patients is complex and involves different decisions and choices during the continuum of care including contraceptive counseling at diagnosis, preventing pregnancy during treatment, contraceptive counseling post treatment and avoiding hormonal contraceptives [8, 14]. Pregnancy during breast cancer treatment is contraindicated as chemotherapy administered during the first trimester results in increased congenital malformations [12, 13, 15]. Most clinicians recommend a delay of at least 2 years after treatment, to allow aggressive breast cancer to become manifest before considering pregnancy, underlining the need for effective, long- acting non-hormonal contraceptive methods [12, 15].

Although breast cancer treatment, especially chemotherapy and hormonal therapy, may have detrimental effects on ovarian function, it does not preclude the possibility of pregnancy during treatment and underscores the need for contraceptive counseling and adherence to avoid an unintended pregnancy [8, 15]. The impact of chemotherapy on fertility is significant. Counseling regarding the possibility of fertility preservation with either ovary or zygote preservation before treatment is important [16]. However, the desire to preserve fertility needs to be balanced against the urgency of cancer treatment as ovarian stimulation will often require a month or two of delay in the starting of systemic treatment [16]. These delays may be significant in poorer resourced health settings where services are not necessarily available in the public sector, and when they are available, waiting times are often long, making fertility preservation options unavailable to many women.

When a woman is diagnosed with breast cancer, initial consultations with the multidisciplinary oncology team focuses predominantly on issues such as the implication of the cancer diagnosis itself, multimodal cancer therapy and coping with a life-threatening disease leaving little room and time for contraceptive counseling [7, 10]. Most oncologists are not experienced in the field of contraception and refer most patients to gynaecologists or family planning clinics [10, 17]. Little is known what happens once women are referred for contraceptive counseling or whether they receive the most suitable contraceptive method or continue with the method.

There is limited data on the contraceptive and fertility counseling needs for women with breast cancer in high- and middle-income countries and no studies in Sub Saharan Africa, including South Africa [4, 8]. This study set out to explore the contraceptive needs and fertility intentions of women of reproductive age (18–49) undergoing breast cancer treatment with the view to support contraceptive and fertility counseling along the continuum of breast cancer care from diagnosis and treatment to survivorship. Health care providers views and experiences of breast cancer patient’s contraceptive and fertility needs were explored to provide further insight into provider-patient counseling needs.

## Methods

Qualitative research methods were used to collect data. In-depth interviews were conducted with women undergoing breast cancer treatment, and health care providers from the Breast Clinic at a large public sector tertiary hospital in Cape Town, South Africa. The population typically served by this large teaching hospital are poorer, under-served patients most of whom do not have private medical insurance. The hospital is also the main referral centre in the Cape Town metropole for women with breast cancer symptoms. The hospital provides breast cancer services to approximately 2.5 million people in the Cape Town area.

The Breast Clinic is an open access, one-stop diagnostic clinic where women may present with a letter from a primary level provider (nurse practitioner or doctor). The breast clinic provides clinical and cytological evaluation with direct referral to an oncologist if breast cytology is positive for malignancy. Newly diagnosed patients are reviewed by a multidisciplinary team including breast surgeons, nurses, oncologists, radiologists, pathologists and social workers. Treatment options include chemotherapy, radiation, breast surgery and genetic counseling. Family planning services are available at the hospital.

### Recruitment and study population

#### Breast cancer patients

Study participants were selected through purposive sampling. In-depth interviews were conducted with 24 women between 2017 and 2018 undergoing different stages of breast cancer treatment. Twenty-nine eligible women were recruited, four did not arrive for their scheduled interview and one woman did not want to wait. Women were recruited with the assistance of the breast clinic staff who helped identify women who were of reproductive age (18–49), diagnosed with breast cancer in the past 5 years and willing to be interviewed about their reproductive and fertility choices. Contraceptive use was not an eligibility criteria. We chose a five-year window to limit recall bias. All women were attending an outpatient appointment at the breast clinic for follow up visits and some were receiving chemotherapy treatment. Eligible participants were identified by the clinic nurse, the research assistant then approached the study participant and further explained the study and invited women to participate in the study.

#### Providers

Prior to the commencement of the study, the first author (JH) met with two clinicians and a genetic counsellor involved in breast cancer treatment and care who then suggested suitable providers to approach. Four in-depth interviews were conducted with health care providers working within the breast clinic and involved in different aspects of treatment and care and included a breast surgeon, oncologist, nursing coordinator and palliative care physician. Interviews were conducted in 2019.

### Data collection

Semi-structured interviews were conducted by female research assistants trained in qualitative research methods in a private space within the hospital. All interviews were audio recorded and conducted in English, isiXhosa or Afrikaans and transcribed and translated into English by an independent transcriber. Interviews were 45–60 min in duration. Data saturation was achieved.

Key areas explored amongst breast cancer patients included; responses to breast cancer diagnosis and what this might mean for future fertility intentions, understandings of suitable contraceptive methods during and after treatment, and future fertility options including fertility preservation. Key areas explored amongst providers included views and opinions around contraceptive and fertility options for women with breast cancer including counseling and intervention needs**.** Interviewers kept fieldnotes which provided added context when reviewing the transcripts. The interview guides were piloted, and changes were made to improve flow and clarity (see Additional files 1 and 2).

### Data analysis

Data were analysed using a thematic analysis approach, in which main themes and categories were identified and analysed within and across data. Initial categories for analysing data were drawn from the interview guide, and then themes and patterns were identified after reviewing the data. The qualitative software package NVivo 12 Pro was used to facilitate sorting and data management.

All transcripts were reviewed by the Principal Investigator and first author (JH) and a preliminary list of codes were developed. The transcripts were coded by a member of the research team (JH) and an independent coder and cross checked for coder variation. The data was then reviewed for major trends, crosscutting themes, and issues for further exploration.

### Ethical considerations

Ethical approval was obtained from the Human Research Ethics Committee, University of Cape Town. Permission to conduct the study was obtained from the tertiary hospital. All study participants provided written informed consent prior to the interview process. Verbal permission was obtained from all study participants prior to digitally recording interviews. Confidentiality and anonymity were ensured. All data were closely controlled and stored in locked files and password protected computer files. Digital recordings were erased once they had been cross checked after data transcription. All women were reimbursed ZAR 100 for their time. Providers were not reimbursed. Study participants had access to counseling services should they require assistance.

## Results

### Participant characteristics

The median age for women (*n* = 24) was 36.5 years, 54% were married or in a long-term relationship, 50% unemployed and 46% had completed high school. Seventy five percent of women (18/24) reported using a contraceptive method, of these 18 women half (9/18) were using the copper T380A IUD, other methods included sterilisation and condoms. More than half of the women (62%) at the time of the interview did not plan to have more children. No women at the time of the interview were known to be pregnant.

Five broad themes were identified linked to the contraceptive and fertility counseling pathways during breast cancer treatment and recovery. These included; i) contraceptive use pre diagnosis; ii) contraception and counseling needs during treatment iii) future fertility intentions iv) fertility preservation options and v) optimal time for contraceptive and fertility counseling.

### Contraceptive use pre diagnosis

We explored patients contraceptive use pre diagnosis as this might have influenced future contraceptive use especially if women were required to change their current contraceptive method. Prior to their breast cancer diagnosis, some women discussed having received limited information about contraceptive method choices from healthcare providers. A breast cancer patient explained:

*Nobody … gave me any advice on contraception they* [healthcare providers] *just said that you have to take a contraceptive and you have to use that one* [3 monthly injectable]. [Age range 41–45, 3 children].

Similarly, another patient recalled limited postpartum contraceptive counseling and method choice which influenced subsequent contraceptive uptake.

*When my daughter was born, they* [nurses] *told me you must go on something before you leave the hospital. So, it wasn’t my choice, they decided for me. So, I went with the injection it was not a good choice. I didn’t know all the pros and cons … I really didn’t want to because I mean why would I want to put something in me that I don’t know what is going inside of me.* [Age range 30–35, 2 children].

### Contraception and counseling during treatment

At diagnosis and during treatment most women reported limited fertility and contraceptive counseling apart from information around changing current hormonal contraception to a non-hormonal method (IUD); the importance of avoiding pregnancy during treatment, and the possible impact of their cancer treatment on future fertility.

### Contraception (non-hormonal methods)

Contraceptive counseling tended to focus on ensuring that patients who were using hormonal methods (injectable and subdermal implant) were provided with the copper T380A IUD prior to treatment highlighted in the excerpts below:

*I was on the 3 months injection* [DMPA] *… they stopped that and then I had to change to the loop* [IUD] … *it prevents the hormones from developing … I can’t use the injection now because of the cancer.* [Age range 46–49, 4 children].

*The doctor* [oncologist] *did discuss contraception because when I was having my next visit for Petogen* [DMPA] *… I was already diagnosed with breast cancer … the doctor said I must stop it because it’s not good for me … they gave me a pamphlet to read at home, they did tell me everything about the IUD.* [Age range 36–40, 2 children].

However, contraceptive counseling and provision at diagnosis and during treatment was not consistent and some women were concerned about the associated risks of an unintended pregnancy. A patient reported receiving limited contraceptive counseling prior to treatment and was concerned about contraceptive safety while undergoing treatment.

*No, I didn’t get advice about family planning … I would have liked to know if I need a family planning, is it safe for me? … I didn’t think about the family planning yet. But I’m going to ask if it’s safe for me to use it.* [Age range 41–45, 1 child].

Related to possible risk, a woman who desired another child considered her options and was fearful of the teratogenic effects of chemotherapy on a pregnancy.

*Before I got diagnosed, we were going to try again to have another child. … But then the doctor also told me that if I fall pregnant while on treatment, they will stop the treatment and obviously I want to have the baby … if I get pregnant even when I’m on treatment it can be that maybe my child can die inside of me if I didn’t know I am pregnant, that’s why you have to be on the loop* [IUD] *or a condom*. [Age range 36–40, 2 children].

### Contraception and counseling: providers

Whilst providers recognised the importance of contraceptive counseling, they noted that their focus as clinicians was on pregnancy prevention during treatment rather than on providing comprehensive contraceptive and fertility counseling during a patient’s treatment trajectory.

A provider concurred with women’s accounts around inconsistent reproductive health counseling, which sometimes resulted in an unintended pregnancy.

*There is a discussion about not falling pregnant with patients, but obviously then things fall through the cracks … it depends on how it goes on the day, who sees you, whether it’s brought up or not. Sometimes this has consequences – we have had a few cases of patients who fell pregnant in between treatments.*

Oncologists main concern was on pregnancy prevention as chemotherapy (especially tamoxifen) was contraindicated in pregnancy. A provider explained that the focus tended to be on preventing a pregnancy during chemotherapy and less on future fertility intentions.

*But fertility is not brought into it so much, it is being mentioned that for the period of your chemotherapy, do not fall pregnant. Not taking it any further than fertility...do you want to have more children in the future, do we have facilities to do banking … that’s not part of the discussion.*

### Future fertility intentions

Women’s future fertility intentions were influenced by numerous factors including their perceived health status, reaching their family size, partner’s desire for more children, suitable time interval before a pregnancy and understandings around the safety of childbearing post cancer treatment.

However, whilst many women did receive information about avoiding pregnancy during treatment and the need to change to a non- hormonal contraceptive method, most women did not receive adequate information on future fertility possibilities or a suitable time in their treatment trajectory to consider childbearing.

An older married woman reflected on her continued desire to have children and sought medical confirmation as to a suitable time to have another child.

*I want to get information* [about childbearing] *from the doctor again because I want to know now after I’m finished with the chemo and I want to find out if I’m healthy now to have more children. My hopes were always to have another child … Before I found out I had cancer … my husband and I were planning to get pregnant, but I must first hear what the doctor says*. [Age range 41–45, 3 children].

Health was an overriding concern and informed decisions about future childbearing.

*I am going to have more children when I see that I am well and when I see that my health has completely improved then I will resume with having children.* [Age range 25–29, 1 child].

However, many women did not want more children having reached their family size or were faced with the reality of a cancer diagnosis and were unsure of their future wellbeing.

*No, I do not want any more children I already have my blessings*. [Age range 36–40, 4 children].

A woman explained that despite wanting another child she and her partner had come to accept that childbearing was not possible due to her breast cancer diagnosis.

*When I mentioned it* [fertility options] *to him* [partner]... *He was also very shocked and disappointed*] *… we didn’t get that chance to discuss it because I just got sick and went for tests and then it’s the cancer … so we didn’t really have that chance to sit and talk with each other about having another baby … I would have loved another one, but unfortunately, I can’t … so for me I’ve got my pigeon pair already, the boy and a girl so for me it’s fine.* [Age range 36–40, 2 children].

### Fertility preservation

We explored whether patients had received any information about fertility preservation procedures prior to treatment. Most patients had not received any information about fertility preservation options, and few were familiar with the concept.

Almost all woman had “*never heard about fertility preservation*”. However, a woman who desired more children explained how she sought information on the internet.

*No they did not discuss fertility options I actually googled it … apparently you can have kids after you had all this radiation and chemo … I actually read up about that* [fertility preservation] *… and then I thought to myself wasn’t I supposed to do that*? [Age range 36–40, 2 children and would like 2 more children].

### Fertility preservation: providers

Providers concurred that fertility preservation options were not discussed and highlighted the difficulties of discussing fertility preservation as it was not easily available in the public sector. Despite there being limited fertility preservation options, especially for younger women, oncologists suggested the possibility of treatment with the gonadotropin-releasing hormone (GnRH) agonist to reduce the risk of chemotherapy induced premature ovarian insufficiency, but cost was an inhibiting factor.

*One of the resources that would help is to have access to ovarian preservation, like your GnRH agonist and then we can give that to younger childbearing women … but there would have to be some sort of subsidy because of the cost.*

### Optimal time for contraceptive and fertility counseling

Providers explored the optimal time in a patient’s treatment trajectory where contraceptive and fertility counseling should occur, suggesting that sexual and reproductive health (SRH) counseling should be integrated into treatment and care. All providers stressed the importance of a “holistic and multidisciplinary” approach to contraceptive and fertility counseling.

*I think contraceptive and fertility counseling needs to be along the continuum of care. I think it’s something that needs to be brought up again and again … and part of a multidisciplinary medical team.*

Whilst women did not directly discuss their sexual health needs, some providers noted that contraceptive and fertility counseling needed to include broader SRH discussions including altered body image and impact of treatment on femininity and motherhood.

*It’s also very important that a person understands that when you do get breast cancer and you’ve gone through all this stuff, your role as a woman has changed … your femininity … So there’s the sense of I am still a woman, I can still have children, I haven’t changed. I think that sense of who I am as a woman needs to be very clearly discussed with patients, now that they have gone through this kind of treatment.*

A health care provider noted that contraception needs to be part of a broader conversation around sexuality and recognised the difficulties as they were not trained in having conversations about more intimate issues.

*It’s not just a conversation about contraception it’s a wider conversation ... it’s sexuality as well … I don’t think you can only just look at contraception. It is the easy, tangible thing, … sexuality is much more complex to deal with … and I don’t think we have trained our health care providers enough in having a sexuality conversation.*

A nurse provider who was involved in counseling patients prior to treatment explained that they did not discuss family planning or contraception in depth nor was it included in the information booklet provided to breast cancer patients.

*The information booklet that I give women with breast cancer doesn’t include contraception and family planning just sexual dysfunction.*

## Discussion

This is the first study undertaken in South Africa exploring the contraceptive and fertility needs and preferences of women diagnosed with breast cancer. Contraceptive counseling focused on discontinuation of hormonal methods and encouraged the uptake of the non-hormonal IUD. Whilst 50% of the women using contraception reported receiving the IUD prior to commencement of treatment, it is unclear whether they continued with their method once treatment was complete.

Unintended pregnancies with the possibility of foetal abnormalities due to adjuvant therapy were reported by providers and underscores the importance of comprehensive contraceptive counseling before and during treatment. In our study women were diagnosed and treated within a relatively short time period, yet women were still at risk of unintended pregnancy and the associated health risks of carrying a pregnancy to term.

Our study is not too dissimilar to research in other countries with more well-resourced oncology services where contraceptive counseling is more likely to occur at diagnosis rather than during treatment and survivorship [4, 10, 11, 18]. However, our study was somewhat different as contraceptive uptake post diagnosis was relatively high amongst study participants compared to other studies in high income settings [4, 7, 19]. The relatively high uptake of the IUD post diagnosis might be different for other non-hormonally mediated cancers and is not reflective of overall IUD uptake in South Africa where the IUD remains under-utilized [20, 21].

Both breast cancer patients and providers reported limited contraceptive counseling throughout the course of treatment highlighting the need for improved information and counseling regarding the impact of treatment on contraceptive and fertility options especially for younger patients desiring more children. Furthermore,

clinicians treating women with breast cancer did not feel they had the requisite skills in providing comprehensive contraceptive and fertility counseling.

Infertility after treatment is a major concern for young women with breast cancer particularly as it relates to opportunities for future childbearing [5, 6, 14]. Research in developed country contexts with more comprehensive programs have highlighted breast cancer patients concerns that their fertility options including fertility preservation were not adequately addressed, exacerbated by a paucity of available information [19, 22, 23]. Providers and patients in our study concurred with these sentiments. Providers felt constrained in discussing fertility preservation options as they were not available or feasible in terms of cost. Fertility preservation options for younger women with breast cancer are complex and requires a multidisciplinary approach including oncologists and reproductive specialists. While there are some well-established options for fertility preservation for women who can afford the cost, many options remain experimental and uncertain [12, 16].

Optimal timing of contraceptive and fertility counseling is important not only in preventing a pregnancy and the associated health consequences but also in providing patients with future fertility options, recognising the personal and emotional experiences of a breast cancer diagnosis. Research has suggested numerous factors are at play at the time of diagnosis, during treatment and into survivorship which could impact on fertility related decision making [4–6, 17]. The timing of counseling played a role in our study and women were not always sure of the best option in terms of future fertility intentions. While not explicitly stated, study participants might have also prioritized survivorship over long-term fertility intentions [4, 17].

Clear guidelines for oncological teams around the need for content, timing and frequency of SRH information has been identified as an often-overlooked area especially for younger breast cancer patients [18, 19]. Associated with the development of these guidelines should be regular SRH educational workshops for multidisciplinary oncology teams to facilitate appropriate counseling to patients. This will facilitate a timely referral pathway between the oncology team to a reproductive health specialist that could provide specialist knowledge, such as fertility preservation options, to assist patients in making informed decisions [8, 10, 11, 16].

An additional area that could be explored in SRH educational workshops includes management of breast cancer diagnosed during pregnancy or treatment**.**

### Limitations

This study has limitations. While the provider sample was small it assisted in triangulating the data and complemented the findings. Breast cancer patients in this study were all seen at a large oncology department within a major teaching hospital, and the findings may not be generalizable to other parts of the country, especially rural areas where access to health services, including cancer care services, might be more challenging.

## Conclusions

Women undergoing breast cancer treatment did not consistently receive counseling on contraception or future fertility options as a part of their care despite being provided with the non- hormonal IUD prior to treatment.

There is a need for improved information and counseling regarding the impact of treatment on contraceptive and feasible fertility options. It is important that health care providers provide timely and ongoing information regarding fertility options and communicate with breast cancer patients about their fertility concerns prior to treatment and throughout the treatment trajectory including survivorship. The development of evidence-based information tools via print and social media platforms could facilitate patient-provider communication and address knowledge gaps especially for younger patients desiring more children. Information tools could also facilitate discussion between not only providers and patients but also wider social networks including family, friends, partners and support groups.

While it is recognized that oncology teams in South Africa are over stretched providing the essentials of cancer care, raising awareness and knowledge about the SRH needs of women with breast cancer amongst all team members, patients and social networks, will catalyze an improvement in contraceptive and fertility support for women during their cancer journey.

## Supplementary information

**Additional file 1.**

**Additional file 2.**

## Data Availability

The datasets used and/or analyzed during the current study are available from the corresponding author upon reasonable request.

## Abbreviations

DMPA: Depot medroxyprogesterone acetate
IUD: Intrauterine device
SRH: Sexual and Reproductive Health
ZAR: South African Rand
