# Supplementary material for: Contraceptive needs and fertility intentions of women with breast cancer in Cape Town, South Africa: a qualitative study
Source: BMC Womens Health. 2020 Oct 6;20:224. doi: 10.1186/s12905-020-01094-3 (PMC7539427; doi:10.1186/s12905-020-01094-3)
Supplement: Supplementary file 1 — Additional file 1. [file 12905_2020_1094_MOESM1_ESM.docx]

**Additional File 1**

**Interview guide: Patients**

**Title: Contraceptive needs and fertility intentions of women with breast cancer in Cape Town, South Africa: a qualitative exploratory study**

**Aim:** identify the contraceptive needs and fertility intentions of women with breast cancer

Key areas: (guide to interviewer)

- Responses to breast cancer diagnosis
- Future fertility intentions prior to diagnosis
- The impact of breast cancer on future fertility intentions
- Impact of breast cancer on efforts to prevent pregnancy
- Understanding of suitable contraceptive methods during and after treatment
- Family or partners response to breast cancer diagnosis, and its implications for future fertility options
- Determine women’s fertility related counselling needs including contraception and fertility planning

**Introduction**

- Introduce yourself and thank the participant for agreeing to be interviewed.
- Explain the purpose of the study
- Before you start the interview, obtain written informed consent and consent to record the interview.
- Prior to putting on the recorder obtain permission to record the interview.

**** Could you tell me what treatment or care you are here for today?**

1. I’d like to start by first asking you some questions around contraception/ family planning. What types of contraception or family planning methods do you know of?

**Probes:**

- *Check individual types depending on her response:* condoms – male & female, the pill, injections ( 2 & 3 monthly) , IUD, implant, male & female sterilization, emergency contraception, condoms
- Have you used any of these methods? If yes, what has been your experiences with these methods ( refer to method they have used by name )
- Probe for side effects, positive or negative experiences
- If not used a method explore why they have not used a method

1. When did you first start using contraception and what or who helped you decide which method to use?

**Probes:**

- Did you speak to anyone in your family about contraception – mother, sister, friends, aunt?
- Did you and your friends discuss or talk about types of contraception?
- What about your partner at the time?
- Family planning clinic, health care provider ?

1. Today you are here for your breast cancer treatment. I’d now like to ask you about having breast cancer. Could you tell me about how you discovered you had breast cancer, and the path you have taken to receiving treatment here at hospital X.

**Probes:**

- Probe distance & time travelled to get to hospital X – how far/ how long it took and where patient is coming from?
- Which was the first medical provider you presented to?
- Which departments have you been referred to?
- Did anything delay your starting chemotherapy or other treatment?

1. What has been your family’s response to your breast cancer diagnosis?

**Probes:**

- Have any family plans had to change?
- How about your partner, specifically?
- Friends

**Can we now talk about breast cancer treatment and the possibility of having children in the future?**

1. How has your illness impacted on your intentions to have children in the future?
2. What is your understanding of the impact of cancer treatment on having children, now and in the future?

**Probes:**

- Have any doctors or nurses spoken to you about this? If yes, what have they told you about having children in the future?
- Do you feel you have been provided with sufficient information?
- Do you want to have more children? If so when?
- How does your partner feel about the impact of cancer treatment on having children?

1. I would like to find out about your experiences with contraception since your breast cancer diagnosis.

*The idea would be to find out if contraception has needed to change, if she has had to start contraception if previously without it, or if she had to stop her contraception (e.g. hormonal) and use barrier methods*

**Probes:**

- Has your contraception had to change since your cancer diagnosis?
- Where have you received your information about contraception and breast cancer treatment? Could you discuss what you have been told by doctors/nurses?
- Could you discuss if you have heard from anywhere else (friends, family media, other sources) ?
- Have you been told there are certain types of contraception you cannot use, now or in the future?
- Have you been to a health care facility to receive contraception? If yes, could you discuss what you were told and did you receive a contraceptive method. If yes, which method did you receive?

1. Apart from the doctors and nurses that are directly involved in your breast cancer treatment, what other support have you received? How did you feel about this support ?

**Probes:**

- Counselling- e.g. genetic counselling, coping with a cancer diagnosis
- Social workers/ counsellors
- Family planning nurses
- Community

1. What other support, in terms of contraception and family planning advice, would you have found helpful since your breast cancer diagnosis?

**Probes:**

- Assess efficacy and efficiency of current services, if any
- Fertility preservation i.e. freezing eggs before treatment
- Assistance in accessing family planning information counselling and care.

1. Explore whether women have any concerns about hormonal contraception (injectable/ oral contraceptive) and its impact on cancer progression or treatment.

Thank you for your time. Are there any other questions that you would like to ask?

We have now come to the end of the interview.
