# Supplementary material for: Contraceptive needs and fertility intentions of women with breast cancer in Cape Town, South Africa: a qualitative study
Source: BMC Womens Health. 2020 Oct 6;20:224. doi: 10.1186/s12905-020-01094-3 (PMC7539427; doi:10.1186/s12905-020-01094-3)
Supplement: Supplementary file 2 — Additional file 2. [file 12905_2020_1094_MOESM2_ESM.docx]

**Additional File 2**

**Interview guide (health care professionals)**

**Title: Contraceptive needs and fertility intentions of women with breast cancer in Cape Town, South Africa: a qualitative exploratory study**

**Introduction**

- Introduce yourself and thank the participant for agreeing to be interviewed.
- Explain the purpose of the study.
- Before you start the interview, obtain written informed consent and consent to record the interview.

**Aim:**

Key areas: (guide to interviewer)

To explore health care providers (surgeons, oncologists and breast cancer nurses) views and experiences of breast cancer patient’s fertility intentions and contraceptive counselling needs.

Key areas to be explored amongst providers will include views and opinions around contraceptive and fertility options for women with breast cancer including counselling and intervention needs.

Introduce yourself and the study.

Record date and site of interview

Allocate a participant number starting with P 01

Prior to putting on the recorder obtain permission to record the interview.

****What is your title specific role here at ….**

**What role do you play in the treatment of women with breast cancer?**

**** How long have you worked in this area?**

1. Could you describe the treatment pathway for breast cancer patients here at hospital X ? From diagnosis to treatment including the types of treatment.

**Probes:**

- How is treatment planning undertaken?

1. I am now going to focus on the study objectives which is to explore the fertility and contraceptive and family planning needs of women with breast cancer.

- **Probes:**
- Could you describe how contraception, family planning and fertility issues are addressed with women diagnosed with breast cancer
- If contraception/ family planning discussed? Where and by whom?

•Are future fertility intentions discussed? Where and by whom?

Who do you think is best placed to discuss contraceptive and family planning issues with breast cancer patients?

- Are patients referred outside the Breast Clinic to receive contraception/family planning advice? If so, are they tracked or is it followed up on by the Breast Clinic?
- Could you discuss if there are any options for fertility preservation or discussions about future fertility intentions.
- How do you think the FP needs of breast cancer patients might differ to other cancer patients ?

1. What has been your experience with the contraceptive/ family planning and fertility concerns of reproductive-aged women with breast cancer?

- Is it an area that you specifically address in your role?
- Is that a topic that the women often address in their treatment or do health professionals need to introduce it?
- If not (1), have you had any informal queries or discussions with patients around family planning, fertility or contraception?
- Have the women you have interacted with planned on having children in the future?
- Have you experienced cases where fertility preservation was used?

1. Where in the treatment cycle do you think would be the most appropriate time to address these women’s contraceptive and fertility concerns and needs?

**Probes:**

- Do you think this an area that needs to be prioritized more?
- Who do you think would be the best person to address it?
- What type of resources do you think you could benefit from having when discussing this topic?
- In your opinion, what are the main barriers in being able to address this topic adequately?

1. What type of information do you think would be most useful for reproductive aged women with breast cancer in terms of contraception and fertility concerns?

**Probes:**

- In your experience are women aware of the treatment’s possible impact on their fertility?
- In your experience do women have an adequate knowledge of the importance of contraception while on treatment and the preferable types of contraception to use when undergoing treatment?
- Have you found that women have adequate knowledge about fertility preservation options available to them?

Thank you for your time. Are there any other questions that you would like to ask?
